# Inverse Association Between Baseline Plasma Selenium Concentrations and Risks of Renal Function Decline in Hypertensive Adults

**DOI:** 10.1093/jn/nxac211

**Published:** 2022-09-09

**Authors:** Youbao Li, Yun Song, Lishun Liu, Xiaobin Wang, Ziyi Zhou, Nan Zhang, Zhuo Wang, Ping Chen, Hanping Shi, Yong Huo, Xiping Xu, Jianping Li

**Affiliations:** Division of Nephrology, Nanfang Hospital, Southern Medical University, Guangzhou, China; National Clinical Research Center for Kidney Disease, Nanfang Hospital, Guangzhou, China; State Key Laboratory of Organ Failure Research, Southern Medical University, Guangzhou, China; Guangdong Provincial Institute of Nephrology, Guangzhou, China; Guangdong Provincial Key Laboratory of Renal Failure Research, Guangzhou, China; Shenzhen Evergreen Medical Institute, Shenzhen, China; Institute for Biomedicine, Anhui Medical University, Hefei, China; Shenzhen Evergreen Medical Institute, Shenzhen, China; Graduate School at Shenzhen, Tsinghua University, Shenzhen, China; Department of Population, Family and Reproductive Health, Johns Hopkins University Bloomberg School of Public Health, Baltimore, MD, USA; Shenzhen Evergreen Medical Institute, Shenzhen, China; Graduate School at Shenzhen, Tsinghua University, Shenzhen, China; Department of Cardiology, Peking University First Hospital, Beijing, China; Key Laboratory of Precision Nutrition and Food Quality, Ministry of Education, Department of Nutrition and Health, College of Food Sciences and Nutritional Engineering, China Agricultural University, Beijing, China; College of Pharmacy, Jinan University, Guangzhou, China; Department of Gastrointestinal Surgery, Beijing Shijitan Hospital, Capital Medical University, Beijing, China; Department of Clinical Nutrition, Beijing Shijitan Hospital, Capital Medical University, Beijing, China; Key Laboratory of Cancer food for special medical purpose (FSMP) for State Market Regulation, Beijing, China; Inspection and Testing Center, Key Laboratory of Cancer FSMP for State Market Regulation, Shenzhen, China; Department of Cardiology, Peking University First Hospital, Beijing, China; Division of Nephrology, Nanfang Hospital, Southern Medical University, Guangzhou, China; National Clinical Research Center for Kidney Disease, Nanfang Hospital, Guangzhou, China; State Key Laboratory of Organ Failure Research, Southern Medical University, Guangzhou, China; Guangdong Provincial Institute of Nephrology, Guangzhou, China; Guangdong Provincial Key Laboratory of Renal Failure Research, Guangzhou, China; Key Laboratory of Precision Nutrition and Food Quality, Ministry of Education, Department of Nutrition and Health, College of Food Sciences and Nutritional Engineering, China Agricultural University, Beijing, China; Department of Cardiology, Peking University First Hospital, Beijing, China

**Keywords:** plasma selenium, folic acid, folate, renal function decline, hypertension

## Abstract

**Background:**

The kidney has the highest level of selenium (Se) in the body, but the role of plasma Se in chronic kidney disease is uncertain.

**Objective:**

We aimed to investigate the longitudinal association between baseline plasma Se and renal function decline in adults with hypertension and to explore possible effect modifiers.

**Methods:**

This was a post hoc analysis of 935 men and women with hypertension aged 40 to 75 years from a folic-acid intervention trial (the China Stroke Primary Prevention Trial) in China. The baseline plasma Se was analyzed both as a continuous variable and as tertiles. The primary outcome was a rapid decline in renal function, defined as a mean decline in the estimated glomerular filtration rate of ≥ 5 mL/(min × 1.73 m^2^) per year.

**Results:**

The median follow-up duration from baseline to outcome was 4.4 years. After multivariate adjustment, there was an inverse association between plasma Se and a rapid decline in renal function (per 10-unit increment; OR: 0.85; 95% CI: 0.73, 0.99). When the baseline plasma Se was assessed as tertiles, compared to the lowest tertile (<74.5 μg/L), a lower trend of the primary outcome was found in the second tertile (74.5 to < 89.4 μg/L; OR: 0.60; 95% CI: 0.34, 1.07) and the highest tertile (89.4 to <150 μg/L; OR: 0.42; 95% CI: 0.22, 0.80; *P*_trend_ = 0.006). Furthermore, the Se-renal association was more pronounced among participants with folic acid treatment or with a higher baseline folate concentration (both *P*_interaction_ values < 0.05).

**Conclusions:**

In this sample of Chinese adults with hypertension, baseline plasma Se concentrations were inversely associated with the risk of renal function decline. The China Stroke Primary Prevention Trial was registered at clinicaltrials.gov as NCT00794885.

## Introduction

Chronic kidney disease (CKD) is a major public health problem worldwide (1). A better understanding of the modifiable risk factors of CKD development could lead to early detection and prevention, helping to alleviate the future burden of CKD and its associated complications. Recently, the potential role of micronutrients in the risk of CKD has received great attention (2–4).

Selenium (Se) is an essential trace element that is incorporated into selenoproteins. Selenocysteine is the key component of several selenoproteins, which have potent antioxidant, antiapoptotic, and anti-inflammatory effects (5). Given that both oxidative stress and inflammation are essential pathophysiologic mechanisms for worsening renal function and that the kidney has the highest levels of Se among all organs, optimizing Se levels may be a potential strategy for preserving renal function (6). Previous animal studies have reported that treatment of Se can alleviate the nephrotoxicity that is induced by lead nitrate (7), cyclophosphamide (8), cisplatin (9), or streptozotocin (10), and Se deficiency has direct effects on renal injury (11). Population studies have also observed that plasma Se levels in nondialyzed CKD patients are lower than those of their healthy counterparts (12, 13). However, longitudinal data on the association between selenium and renal function remain limited (14). Few previous studies have comprehensively investigated potential modifiers of the relation between Se and renal function.

To address the above knowledge gaps, this current study aimed to examine the association of baseline plasma Se with the risk of renal function decline and to identify potential effect modifiers among adult patients with hypertension, a high-risk population for developing CKD (15).

## Methods

### Study participants and design

This was a post hoc analysis of 935 hypertensive adults from the China Stroke Primary Prevention Trial (CSPPT), a folic acid intervention trial to prevent stroke. The study design and major results of the CSPPT (registered at clinicaltrials.gov as NCT00794885) (16) and the renal substudy of the CSPPT have been described in detail previously (2). Briefly, the CSPPT was a randomized, double-blinded, actively controlled trial that was conducted from May 2008 to August 2013 in 32 communities in China. Eligible participants were men and women aged 45 to 75 years who had hypertension, defined as seated, at-rest systolic blood pressure (SBP) ≥ 140 mmHg or diastolic blood pressure (DBP) ≥ 90 mmHg at both the screening and recruitment visit, or who were using antihypertensive medication. The major exclusion criteria included a history of physician-diagnosed stroke, myocardial infarction (MI), heart failure, coronary revascularization, congenital heart disease, and current supplementation by folic acid.

A total of 20,702 eligible participants were randomly assigned, in a 1:1 ratio, to 1 of 2 treatments: *1*) a daily oral dose of 1 tablet containing 10 mg of enalapril and 0.8 mg of folic acid (the enalapril–folic acid group); or *2*) a daily oral dose of 1 tablet containing 10 mg of enalapril only (the enalapril group). The renal substudy (*n* = 15,014) enrolled CSPPT participants from 20 communities in Jiangsu province, with the exclusion of individuals with an estimated glomerular filtration rate (eGFR) < 30 mL/(min × 1.73 m^2^) or with missing eGFR values at baseline. Participants were followed up every 3 months. At each visit, participant blood-pressure and pulse measurements were obtained. Triplicate measurements on the same arm were taken, with at least 2 minutes between readings. The mean SBP and mean DBP were calculated from the 3 independent measures and used in the analyses. Before the study ended, an exit visit was conducted for blood sample collection and an assessment of renal outcomes.

The renal substudy of the CSPPT suggested that folic acid treatment can significantly delay the progression of CKD by 55% among hypertensive patients with mild to moderate CKD (2). This current study (*n* = 935) included those participants from the renal substudy of the CSPPT who had data on Se measurements and with plasma Se < 150 μg/L, as well as those who had complete data on creatinine at both the baseline and exit visits (**Supplemental Figure 1**).

The parent study (CSPPT) was approved by the Ethics Committee of the Institute of Biomedicine, Anhui Medical University, Hefei, China (FWA assurance number: FWA00001263). All participants provided written, informed consent.

### Laboratory assays

Blood and spot-urine samples were collected from the participants at both the baseline and the exit visits. Methylenetetrahydrofolate reductase (*MTHFR)* C677T (rs1801133) polymorphisms were detected on an ABI Prism 7900HT sequence detection system (Life Technologies) using the TaqMan assay. Serum folate and vitamin B12 were measured by a commercial laboratory using a chemiluminescent immunoassay (New Industrial). CVs for the assays of folate and B12 were 2.9% and 2.3%, respectively. Serum creatinine, uric acid, total homocysteine (tHcy), total cholesterol (TC), and fasting glucose were measured using automatic clinical analyzers (Beckman Coulter) at the core laboratory of the National Clinical Research Center for Kidney Disease, Guangzhou, China. Specifically, serum creatinine was measured using an enzymatic assay that had been calibrated to be isotope dilution MS traceable. The CV for the assay was 1.4%. The eGFR was calculated using the Chronic Kidney Disease Epidemiology Collaboration equation (17). Proteinuria was captured on the basis of spot-urine samples by the dipstick test, using an automated urine analyzer (Dirui-H100) (18). Plasma Se was measured by inductively coupled plasma mass spectrometry (ICP-MS) using Thermo Fisher iCAP Q ICP-MS in a commercial lab (Beijing DIAN Medical Laboratory). Both intra-assay and inter-assay CVs of duplicate samples (randomly placed among the study samples) were calculated. The intra-assay CV of Se ranged from 1.42% to 6.13%, whereas the inter-assay CV of Se was 4.67%.

### Study outcomes

The primary outcome was a rapid decline in renal function, defined as a mean decline in eGFR of 5 mL/(min × 1.73 m^2^) or more per year.

The secondary outcome was the development or progression of CKD, defined as any of the following: *1*) a decrease in the eGFR of 30% or more and to a level of less than 60 mL/(min × 1.73 m^2^) at the exit visit if the baseline eGFR was 60 mL/(min × 1.73 m^2^) or more; *2*) a decrease in the eGFR of 50% or more at the exit visit if the baseline eGFR was less than 60 mL/(min × 1.73 m^2^); or *3*) end-stage renal disease, defined as an eGFR < 15 mL/(min × 1.73 m^2^) or the need for dialysis (2).

### Other definitions

BMI was calculated as weight divided by height squared (kg/m^2^). Time-averaged blood pressure (BP) over the course of treatment was calculated for each participant using all postbaseline measurements up to the last visit. Participants with an eGFR < 60 mL/(min × 1.73 m^2^) and/or proteinuria at baseline were classified as having CKD (2). Having proteinuria was defined as having a urine protein reading of trace or more. Having diabetes mellitus was defined as having a history of diabetes, a fasting glucose level of 7 mmol/L or more, or undergoing glucose-lowering therapy at baseline. Having hypercholesteremia was defined as having a TC of 6.2 mmol/L or more. A status of currently smoking was defined as smoking at least 1 cigarette per day or smoking > 18 packs in the prior year.

### Statistical analyses

Of the 935 participants, there were missing data on serum folate (*n* = 11), vitamin B12 (*n* = 11), and urine protein status (*n* = 33) at baseline. As a categorical variable, missing values for the urine protein status were replaced by a new category of “missing,” creating a new categorical variable for the urine protein status (absent, present, or missing) that was used in the subsequent analyses. When stratified analyses were conducted on serum folate or vitamin B12, any study participants with missing data on folate or vitamin B12 were excluded from the analysis, reducing the sample size to 924.

Participant characteristics were presented as means ± SDs for continuous variables and proportions for categorical variables, according to the tertiles of baseline plasma Se. Differences in baseline characteristics by tertiles of plasma Se were compared using ANOVA tests or chi-square tests, accordingly.

ORs and 95% CIs of the study outcomes were estimated by modeling plasma Se both as a continuous variable and as tertiles using logistic regression models, without or with adjustment of covariables. Covariables were included in the models as continuous (per 1-unit increment) or categorical, as appropriate, including age (year), sex (male compared with female), eGFR [mL/(min × 1.73 m^2^)], treatment group (enalapril–folic acid group compared with enalapril group), the presence of proteinuria, hypercholesteremia (TC ≥ 6.2 mmol/L), BMI (kg/m^2^), *MTHFR* C677T polymorphisms (CC, CT, and TT), SBP (mmHg), the presence of diabetes, smoking status (ever smokers compared with never smokers) at baseline, and time-averaged SBP during the follow-up period (mmHg).

In addition, possible modifications of the association between plasma Se (per 10-unit increment) and a rapid decline in renal function were also assessed for the following variables: age (≥60 compared with <60 years), sex, the presence of CKD (present compared with absent), the presence of diabetes (present compared with absent), treatment group (enalapril–folic acid compared with enalapril), serum folate (≥9.0 compared with <9.0 ng/mL), serum vitamin B12 (≥280 compared with <280 pmol/L), and serum tHcy (<10, 10 to <15, and ≥15 μmol/L), by including interaction terms (plasma Se, per 10-unit increment; × factor) into the multivariate-adjusted models.

A 2-tailed *P* value < 0.05 was considered to be statistically significant in all analyses. R software, version 3.4.2 (R Foundation) was used for all statistical analyses.

## Results

### Characteristics of the study participants

As illustrated in Supplemental Figure 1, a total of 935 participants were included in the final analysis. The baseline characteristics of participants included in this analysis did not differ from those of participants not included, except for baseline BP (**Supplemental Table 1**). The mean age of the participants was 59.6 years, and 38.0% were males. The general characteristics of the participants included in this analysis are presented according to the tertiles of plasma Se. Participants with higher plasma Se concentrations were more likely to have higher levels of fasting glucose, TC, and vitamin B12, but lower tHcy levels (**Table 1**).

**TABLE 1:** Characteristics of participants by tertiles of plasma selenium^1^

	Plasma selenium tertiles, μg/L			
	T1 (<74.5)	T2 (74.5 to <89.4)	T3 (89.4 to <150)	*P*
Participants, *n*	312	309	314	
At baseline				
Male, *n* (%)	112 (35.9)	118 (38.2)	125 (39.8)	0.599
Age, y	60.3 ± 7.8	59.1 ± 7.5	59.4 ± 7.6	0.138
BMI, kg/m^2^	25.5 ± 3.6	25.7 ± 3.8	25.6 ± 3.5	0.713
*MTHFR* 677TT, *n* (%)	88 (28.2)	81 (26.2)	78 (24.8)	0.631
Treatment group, *n* (%)	—	—	—	0.735
Enalapril group	157 (50.3)	153 (49.5)	165 (52.5)	
Enalapril–folic acid group	155 (49.7)	156 (50.5)	149 (47.5)	
Current smoking, *n* (%)	61 (19.6)	76 (24.6)	68 (21.7)	0.312
Diabetes mellitus, *n* (%)	32 (10.3)	39 (12.6)	51 (16.2)	0.081
CKD, *n* (%)	37 (11.9)	31 (10.0)	36 (11.5)	0.748
SBP, mmHg	167 ± 19.3	167 ± 20.5	166 ± 20.4	0.790
DBP, mmHg	93.5 ± 11.6	94.4 ± 11.9	95.1 ± 12.3	0.236
Laboratory results				
Serum total cholesterol, mmol/L	5.4 ± 1.1	5.6 ± 1.1	5.9 ± 1.1	<0.001
Serum fasting glucose, mmol/L	5.7 ± 1.4	5.8 ± 1.5	6.2 ± 2.1	<0.001
Serum tHcy, μmol/L	16.1 ± 10.9	14.3 ± 7.6	13.2 ± 6.6	<0.001
Serum folate, ng/mL	8.1 ± 3.5	7.3 ± 2.7	7.6 ± 2.9	0.007
Serum vitamin B12, pmol/L	277 ± 93.5	292 ± 91.4	322 ± 127.6	<0.001
eGFR, mL/(min × 1.73 m^2^)	92.8 ± 13.2	94.4 ± 12.2	94.3 ± 12.8	0.242
Serum uric acid, μmol/L	291 ± 74.5	300 ± 83.9	299 ± 79.2	0.247
Plasma selenium, μg/L	65.9 ± 6.2	81.7 ± 4.2	104 ± 12.8	<0.001
Medication use, *n* (%)				
Antihypertensive drugs	156 (50.0)	142 (46.0)	163 (51.9)	0.316
Lipid-lowering drugs	1 (0.3)	3 (1.0)	1 (0.3)	0.438
Glucose-lowering drugs	2 (0.6)	4 (1.3)	9 (2.9)	0.075
Antiplatelet drugs	16 (5.1)	13 (4.2)	16 (5.1)	0.831
During follow-up or at exit				
Time-averaged SBP during follow-up	139 ± 10.8	139 ± 10.4	140 ± 10.2	0.542
Time-averaged DBP during follow-up	83.1 ± 7.3	83.0 ± 6.9	83.8 ± 7.1	0.287
eGFR at exit, mL/(min × 1.73 m^2^)	86.4 ± 16.7	89.4 ± 14.6	89.9 ± 15.0	0.008

1 For continuous variables, values are presented as means ± SDs. CKD, chronic kidney disease; DBP, diastolic blood pressure; eGFR, estimated glomerular filtration rate; *MTHFR*, methylenetetrahydrofolate reductase; SBP, systolic blood pressure; T, tertile; tHcy, total homocysteine.

In addition, during the treatment period, the frequencies in use of concomitant calcium channel blockers, diuretics, antiplatelet drugs, and lipid-lowering drugs were similar across all plasma Se tertiles. However, participants with higher Se levels also had a higher frequency of glucose-lowering drug use (**Supplemental Table 2**).

### Association between plasma Se and renal outcomes

During the median treatment duration of 4.4 years (IQR: 4.2–4.6 years), a rapid decline in renal function and the development or progression of CKD occurred in 72 (7.7%) and 33 (3.5%) participants, respectively.

**FIGURE 1:**
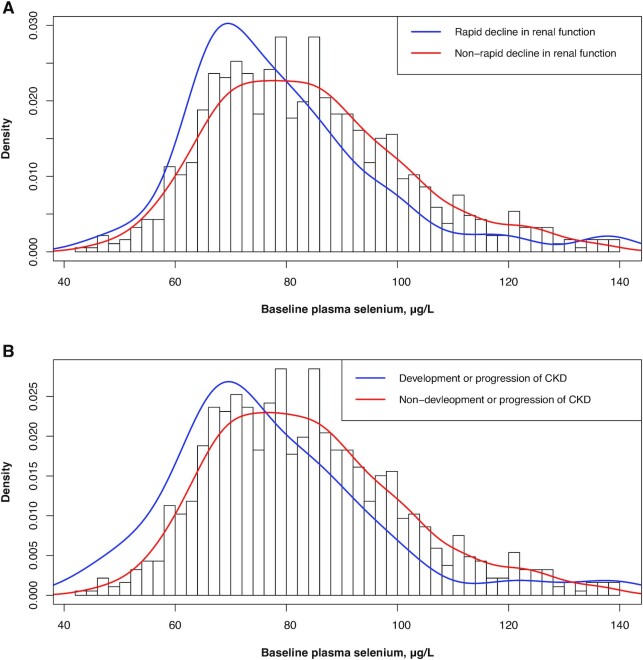
The frequency distribution of baseline plasma selenium in 935 participants by outcome status: (A) rapid decline in renal function and (B) development or progression of CKD. CKD, chronic kidney disease.

As shown in **Figure 1**, participants who experienced a rapid decline in renal function or the development or progression of CKD showed a baseline plasma Se distribution that shifted towards the left tail compared to their counterparts. Consistently, there was an inverse association between plasma Se and the risk of a rapid decline in renal function (per 10-unit increment; multivariate-adjusted OR: 0.85; 95% CI: 0.73, 0.99) and the development or progression of CKD (per 10-unit increment; multivariate-adjusted OR: 0.81; 95% CI: 0.65, 1.01). When the baseline plasma Se values were analyzed as tertiles, compared to the lowest tertile (<74.5 μg/L), lower trends of rapid declines in renal function were found in the second tertile (74.5 to < 89.4 μg/L; OR: 0.60; 95% CI: 0.34, 1.07) and the highest tertile (89.4 to <150 μg/L; OR: 0.42; 95% CI: 0.22, 0.80; *P*_trend_ = 0.006).

Similar results were found for the association of plasma Se and the risk of the development or progression of CKD (**Table 2**).

**TABLE 2:** The association between plasma selenium and renal outcomes in hypertensive adults from a folate supplementation trial^1^

		Crude model	Multivariate adjusted^2^	
Plasma selenium, μg/L	Events/participants, *n/n* (%)	OR (95% CI)	OR (95% CI)	*P*
Rapid decline in renal function				
Per 10-unit increment	72/935 (7.7)	0.83 (0.72–0.97)	0.85 (0.73–0.99)	0.042
Tertiles				
T1 (<74.5)	35/312 (11.2)	Ref (1.0)	Ref (1.0)	
T2 (74.5 to <89.4)	22/309 (7.1)	0.61 (0.35–1.06)	0.60 (0.34–1.07)	0.081
T3 (89.4 to <150)	15/314 (4.8)	0.40(0.21–0.74)	0.42(0.22–0.80)	0.009
*P*_trend_		0.003	0.006	
Development or progression of CKD				
Per 10-unit increment	33/935 (3.5)	0.79 (0.63–0.98)	0.81 (0.65–1.01)	0.064
Tertiles				
T1 (<74.5)	18/312 (5.8)	Ref (1.0)	Ref (1.0)	—
T2 (74.5 to <89.4)	9/309 (2.9)	0.49 (0.22–1.11)	0.55 (0.24–1.28)	0.164
T3 (89.4 to <150)	6/314 (1.9)	0.32 (0.12–0.81)	0.32 (0.12–0.85)	0.022
*P*_trend_		0.011	0.017	

1 CKD, chronic kidney disease; eGFR, estimated glomerular filtration rate; *MTHFR*, methylenetetrahydrofolate reductase; SBP, systolic blood pressure; T, tertile.

2 Adjusted for age, sex, eGFR, treatment group, BMI, *MTHFR* C677T polymorphisms, the presence of proteinuria, hypercholesteremia, SBP, the presence of diabetes, smoking status at baseline, and time-averaged SBP during the follow-up period.

### Exploratory subgroup analyses

**FIGURE 2:**
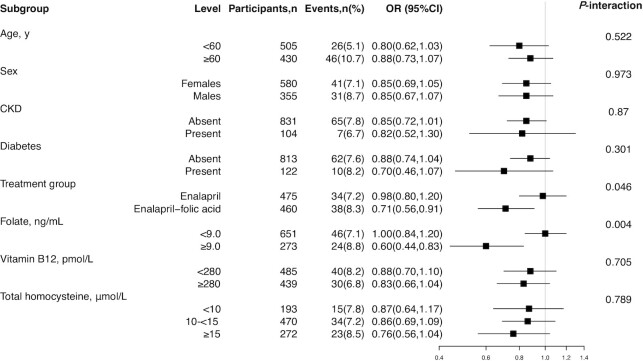
Forest plot of subgroup analyses on the association between plasma selenium (per 10-unit increment) and the risk of rapid decline in renal function in hypertensive adults from a folate supplementation trial. Adjusted, if not stratified, for age, sex, eGFR, treatment group, BMI, *MTHFR* C677T polymorphisms, the presence of proteinuria, hypercholesteremia, SBP, the presence of diabetes, smoking status at baseline, and time-averaged SBP during the follow-up period. CKD, chronic kidney disease; eGFR, estimated glomerular filtration rate; MTHFR, methylenetetrahydrofolate reductase; SBP, systolic blood pressure.

Stratified analyses were performed to assess the association between plasma Se (per 10-unit increment) and the rapid decline in renal function in various subgroups (**Figure 2**). A stronger inverse relation of plasma Se with a rapid decline in renal function was observed in participants in the enalapril–folic acid group (OR: 0.71; 95% CI: 0.56, 0.91) compared to those in the enalapril group (OR: 0.98; 95% CI: 0.80, 1.20; *P*_interaction_ = 0.046). Similarly, a significant, inverse association between Se and renal function decline was also stronger in participants with high baseline folate levels [≥9.0 ng/mL (OR: 0.60; 95% CI: 0.44, 0.83) compared with <9.0 ng/mL (OR: 1.00; 95% CI: 0.84, 1.20); *P*_interaction_ = 0.004; Figure 2]. Other variables, including age, sex, the presence of CKD, the presence of diabetes, vitamin B12 level, or tHcy level at baseline, did not appear to modify the association between Se and renal function (Figure 2).

## Discussion

Our study demonstrated a significant, inverse association between plasma Se and the subsequent risk of renal function decline in hypertensive patients. Furthermore, folate was a significant modifier for the relation of plasma Se and renal function. The inverse association was more pronounced in participants receiving folic acid treatment or with higher baseline folate levels (i.e., ≥9.0 ng/mL). However, it should be noted that given the existing sample size, the positive findings of the interaction test could be due to chance.

### Previous longitudinal studies on the association between Se and renal function

The association between Se and renal function remains uncertain. Farhadnejad et al. (14) reported no significant association between Se intake, based on an FFQ, and the risk of incident CKD. Of note, the Se content of food varies widely, and is mainly a reflection of soil conditions (19). As such, the calculation of Se intake with food composition databases is perhaps inaccurate. In contrast, blood Se is usually considered to be a reliable biomarker for selenium status (20). Our study provided an opportunity to assess the temporal and dose-response relations between plasma Se and renal function decline in a general, treated hypertensive population. This study not only had data on plasma Se concentrations, but also had all the pertinent clinical information and laboratory measurements.

In support of our findings, a randomized, double-blind, placebo-controlled, phase-II, crossover study of 74 patients with leukemias and lymphomas (LL) and solid tumors (ST) reported that although there was no statistically significant difference in serum creatinine levels during either placebo or Se supplementation, 36.8% of LL patients (*n* = 7) presented serum creatinine levels above normal values during placebo intake, which decreased to 26.3% of LL patients (*n* = 5) during Se intake (21). More recently, Alehagen et al. (22) found that compared with placebo, supplementation with selenium and coenzyme Q10 resulted in significantly improved renal function in 215 elderly with low selenium (mean, 67 μg/L) after a follow-up of 5.1 years. However, this study may only be hypothesis generating due to its small sample size. Furthermore, as the intervention arm included both selenium (200 μg/day) and coenzyme Q10 (200 mg/day), the effective element was unclear. Therefore, the effect of Se supplementation on renal function should be further investigated in future randomized trials with large sample sizes among widespread populations with Se deficiency.

### The potential mechanisms on the association between Se and renal function

Whilst the exact mechanisms underlying the inverse association of Se and renal function declines remains uncertain, it has been found that selenium exerts antioxidation, anti-inflammatory, and antiapoptotic functions, thus reducing kidney injuries and alleviating renal function declines, in animals (7–11, 23–25). For instance, selenoproteins, such as glutathione peroxidase 3 and selenoprotein P, may prevent the oxidative modification of lipids and inhibit platelet aggregation (5). In addition, selenium inhibited the activation of NOD-like receptor thermal protein domain associated protein 3 (NLRP3) inflammasome to attenuate lead-induced inflammatory damage in chicken kidneys (23). Several animal studies also observed that treatment with selenium alleviated kidney cell apoptosis via the PI3K/AKT, Nrf2/Keap1 or P38/JNK/ERK pathways (24, 25). These findings may partly help to explain how higher selenium levels reduce the risk of decreased kidney functions.

### Strengths of our study

Our study has made new contributions to the field. First, to our knowledge, this is the first study of its kind in Chinese adults with hypertension, a high-risk population for developing CKD. It showed an inverse association between plasma Se and the risk of declines in renal functions. If further confirmed, these findings could have important clinical and public health implications. Second, this study offers important new insights regarding the interplay of folate and Se on renal functions. We found that folate status was a significant modifier: a stronger inverse association between Se and renal function declines was found among participants receiving folic acid treatment or with baseline folate ≥ 9.0 ng/mL. This finding is biologically plausible. It has been reported that in addition to its effect on tHcy levels, folate has direct antithrombotic and antioxidant effects, and can ameliorate endothelial dysfunction and nitrate tolerance (26). While most previous studies have examined micronutrients individually, in real life a person is simultaneously exposed to many nutrients. Our findings underscore the need for future studies to carefully consider a Se-folate interaction on renal function and, perhaps, interactions of other micronutrients not yet examined in this study.

### Limitations of our study

Our study also has several limitations. First, as an observational study, although a number of covariates have been adjusted for in the regression models, we cannot exclude the possibility of residual confounding from other unmeasured or unknown factors, such as detailed information on antioxidant dietary intakes, dietary habits, and physical activity. Second, Se was only measured at a single time point. More frequent measurements of Se would have provided more information. Third, due to a lack of relatively high levels of Se in this study population, we were unable to further investigate the potentially U-shaped relationship of Se with renal function. Moreover, this sample size was not large enough to explore the 3-way interactions of selenium, folate, and tHcy on renal outcomes. Fourth, the study population consisted of hypertensive adults from China; the generalizability of the results to a general population or to other ethnicities remains to be verified. Owing to the study limitations, confirmation of our findings in an independent study is necessary.

### Conclusions

Our study showed an inverse association of baseline plasma Se levels with the risk of renal function decline in adults with hypertension, especially among those receiving folic acid treatment or with higher baseline folate levels, suggesting a synergistic effect of Se and folate in preserving renal function. Our findings warrant additional investigation.

## Supplementary Material

nxac211_Supplemental_File

## Data Availability

Data described in the manuscript, code book, and analytic code will be made available from the corresponding author on request, after the request is submitted and formally reviewed and approved by the Ethics Committee of the Institute of Biomedicine, Anhui Medical University, and the Ethics Committee of Nanfang Hospital.

## Notes

This work was supported by the National Natural Science Foundation of China (82000691); the Science and Technology Planning Project of Guangzhou, China (202102021155); and the Outstanding Youths Development Scheme of Nanfang Hospital, Southern Medical University (2019J004).

Author disclosures: Dr. Youbao Li reports grants from the National Natural Science Foundation of China, the Science and Technology Planning Project of Guangzhou, China, and the Outstanding Youths Development Scheme of Nanfang Hospital, Southern Medical University. All other authors report no conflicts of interest.

The funders had no role in the design and conduct of the study (data collection, management, analysis, and interpretation), the preparation, review, approval of the manuscript, or the decision to submit the manuscript for publication. This work was supported by the National Natural Science Foundation of China [82000691]; the Science and Technology Planning Project of Guangzhou, China [202102021155]; and the Outstanding Youths Development Scheme of Nanfang Hospital, Southern Medical University [2019J004].

Supplemental Figure 1 and Supplemental Tables 1 and 2 are available from the “Supplementary data” link in the online posting of the article and from the same link in the online table of contents at https://academic.oup.com/jn/.

Abbreviations used: BP, blood pressure; CKD, chronic kidney disease; CSPPT, China Stroke Primary Prevention Trial; DBP, diastolic blood pressure; eGFR, estimated glomerular filtration rate; ICP-MS, inductively coupled plasma mass spectrometry; LL, leukemias and lymphomas; MI, myocardial infarction; MTHFR, methylenetetrahydrofolate reductase; SBP, systolic blood pressure; Se, selenium; ST, solid tumors; TC, total cholesterol; tHcy, total homocysteine.
