# Supplementary material for: Inverse Association Between Baseline Plasma Selenium Concentrations and Risks of Renal Function Decline in Hypertensive Adults
Source: J Nutr. 2022 Sep 9;152(12):2754–60. doi: 10.1093/jn/nxac211 (PMC9839988; doi:10.1093/jn/nxac211)
Supplement: nxac211_Supplemental_File [file nxac211_supplemental_file.docx]

On-line Supplementary Material

Li Y, et al. Inverse association between baseline plasma selenium concentrations and risk of renal function decline in hypertensive adults

**
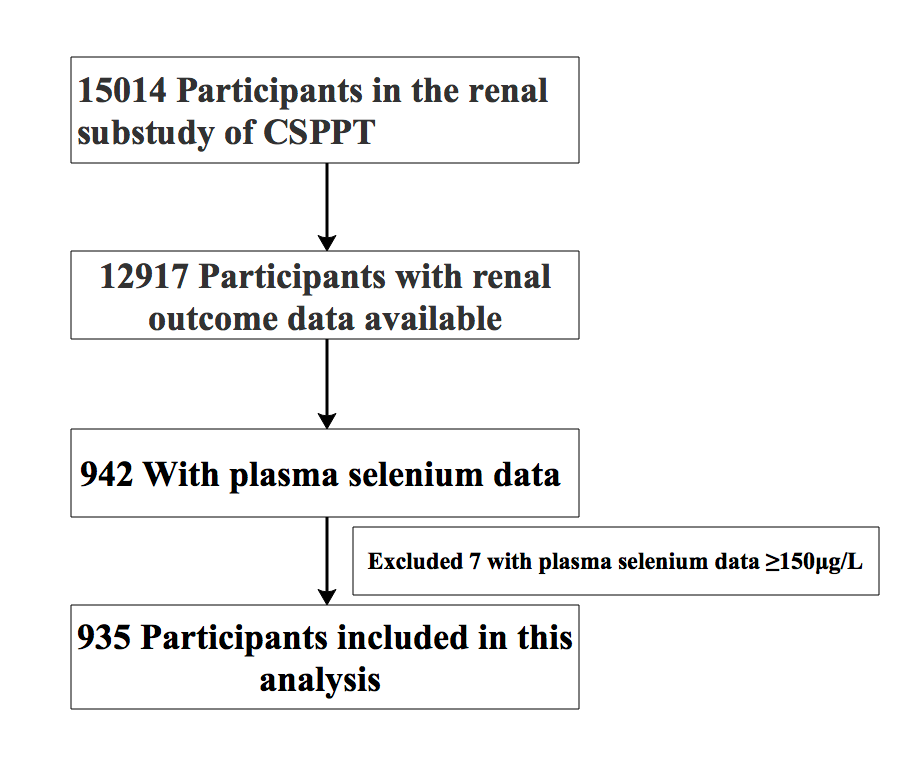
**

**Supplementary Figure 1. Flow chart of the study participants**

On-line Supplementary Material

Li Y, et al. Inverse association between baseline plasma selenium concentrations and risk of renal function decline in hypertensive adults

**Supplementary Table 1. Baseline characteristics of participants of the renal sub-study of the CSPPT included or not included in this analysis^1^**

|  | **Participants of the renal sub-study of the CSPPT** | | ***P*** |
| --- | --- | --- | --- |
|  | **Not-included in this analysis** | **Included in this analysis** |  |
| **Participants, n** | 11982 | 935 |  |
| **Male, n (%)** | 4614(38.5) | 355(38.0) | 0.770 |
| **Age, y** | 59.6±7.5 | 59.6±7.6 | 0.896 |
| **BMI, kg/m^2^** | 25.7±3.5 | 25.6±3.6 | 0.339 |
| ***MTHFR* 677 TT, n (%)** | 3202(26.7) | 247(26.4) | 0.869 |
| **Treatment group, n (%)** |  |  | 0.712 |
| Enalapril group | 6005(50.1) | 475(50.8) |  |
| Enalapril-folic acid group | 5977(49.9) | 460(49.2) |  |
| **Current smoking****, n (%)** | 2644(22.1) | 205(21.9) | 0.948 |
| **Diabetes mellitus, n (%)** | 1639(13.7) | 122(13.0) | 0.623 |
| **CKD, n (%)** | 1300(10.8) | 104(11.1) | 0.838 |
| **Blood pressure, mmHg** |  |  |  |
| Baseline SBP | 169±21.0 | 167±20.0 | 0.024 |
| Baseline DBP | 95.4±11.9 | 94.3±12.0 | 0.010 |
| Time-averaged SBP during follow-up | 140±11.0 | 139±10.4 | 0.874 |
| Time-averaged DBP during follow-up | 83.5±7.3 | 83.3±7.1 | 0.365 |
| **Laboratory results** |  |  |  |
| Serum total cholesterol, mmol/L | 5.7±1.2 | 5.6±1.1 | 0.082 |
| Serum fasting glucose, mmol/L | 6.1±1.8 | 5.9±1.7 | 0.088 |
| Serum tHcy, μmol/L | 14.6±8.9 | 14.5±8.6 | 0.746 |
| Serum folate, ng/mL | 7.7±3.3 | 7.7±3.1 | 0.851 |
| Serum vitamin B-12, pmol/L | 299±117.8 | 297±107.2 | 0.577 |
| eGFR, mL/(min· 1.73m^2^) | 94.1±12.8 | 93.8±12.8 | 0.560 |
| Serum uric acid, μmol/L | 293±78.9 | 297±79.3 | 0.234 |
| **Medication use, n (%)** |  |  |  |
| Antihypertensive drugs | 5990(50.0) | 461(49.3) | 0.711 |
| Lipid-lowering drugs | 109(0.9) | 5(0.5) | 0.318 |
| Glucose-lowering drugs | 222(1.9) | 15(1.6) | 0.675 |
| Antiplatelet drugs | 462(3.9) | 45(4.8) | 0.173 |

**^1^**All the data are baseline values, except for blood pressure. For continuous variables, values are presented as mean (SD).

**Abbreviations:** BMI, body mass index; CKD, chronic kidney disease; DBP, diastolic blood pressure; eGFR, estimated glomerular filtration rate; *MTHFR*, methylenetetrahydrofolate reductase; SBP, systolic blood pressure; and tHcy, total homocysteine.

On-line Supplementary Material

Li Y, et al. Inverse association between baseline plasma selenium concentrations and risk of renal function decline in hypertensive adults

**Supplementary Table 2. Concomitant medication usage during follow-up by tertiles of plasma selenium in hypertensive adults from a folate intervention trial^1^**

| Concomitant medication use, n/participants (%) | Plasma selenium tertiles, μg/L | | | *P* |
| --- | --- | --- | --- | --- |
|  | T1(<74.5) | T2(74.5-<89.4) | T3(89.4-<150) |  |
| **Antihypertensive drugs** |  |  |  |  |
| Calcium channel blockers | 247/312 (79.2) | 256/309 (82.8) | 253/314(80.6) | 0.501 |
| Diuretics | 208/312 (66.7) | 187/309(60.5) | 187/314(59.6) | 0.138 |
| **Glucose-lowering drugs** | 1/312 (0.3) | 5/309(1.6) | 9/314(2.9) | 0.040 |
| **Antiplatelet drugs** | 3/312 (1.0) | 5/309(1.6) | 2/314(0.6) | 0.480 |
| **Lipid-lowering drugs** | 1/312 (0.3) | 0/309(0.0) | 0/314(0.0) | 0.368 |

^1^Regular concomitant medication was defined as 180 or more cumulative days of taking the drug of interest.
